# Fast ensemble representations for abstract visual impressions

**DOI:** 10.1038/ncomms13186

**Published:** 2016-11-16

**Authors:** Allison Yamanashi Leib, Anna Kosovicheva, David Whitney

**Affiliations:** 1 University of California Berkeley, Whitney Lab, 3210 Tolman Hall, Berkeley, California 94720, USA; 2 Northeastern University, 360 Huntington Ave, Boston, Massachusetts 02115, USA

## Abstract

Much of the richness of perception is conveyed by implicit, rather than image or feature-level, information. The perception of animacy or lifelikeness of objects, for example, cannot be predicted from image level properties alone. Instead, perceiving lifelikeness seems to be an inferential process and one might expect it to be cognitively demanding and serial rather than fast and automatic. If perceptual mechanisms exist to represent lifelikeness, then observers should be able to perceive this information quickly and reliably, and should be able to perceive the lifelikeness of crowds of objects. Here, we report that observers are highly sensitive to the lifelikeness of random objects and even groups of objects. Observers' percepts of crowd lifelikeness are well predicted by independent observers' lifelikeness judgements of the individual objects comprising that crowd. We demonstrate that visual impressions of abstract dimensions can be achieved with summary statistical representations, which underlie our rich perceptual experience.

## Abstract (web summary)

Perceiving objects as lifelike is an inferential process but whether it occurs quickly and how it applies to groups of objects is not well understood. Here the authors show that observers' percepts of crowd lifelikeness are fast and represent the average of the individual objects comprising that crowd.

## Graphical abstract

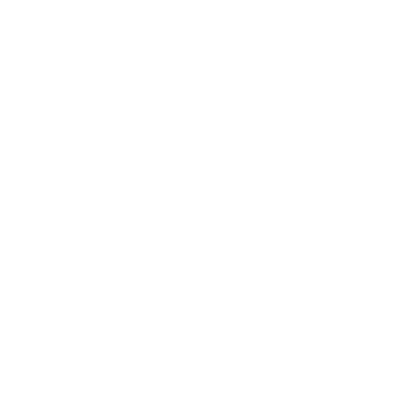

Even at first glance, observers report that their visual perception seems rich and complete^1^. At least some of this richness may be supported by ensemble or summary statistical representations. Prior work has shown that ensembles support gist level interpretations of scenes^2^; however, until now, ensemble perception has only been demonstrated for explicit visual dimensions such as size, orientation, motion and faces^3^,^4^,^5^. What mechanism underlies the visual experiences that go beyond simple visual features, textures and explicit dimensions in scenes? Observers can—at least cognitively—interpret abstract content from scenes, including emotional and social information^6^,^7^. For example, observers can report the inferred emotional pain from a photograph of a serious car accident, or the implied sense of urgency in a photograph of a panicking crowd^8^. One striking abstract impression that observers report is animacy, or liveliness, of an object or scene^9^,^10^,^11^. These sorts of high-level perceptual impressions are based on visual information but are not directly available from the image content itself (at least not in a well-defined or straightforward way)^12^. Researchers often assume that these high-level impressions must be cognitive: requiring attention to contextual information, deliberation about meaning, observer specific learning, or other potentially slow or serial processes^13^,^14^,^15^. An alternative possibility is that these high-level visual impressions, such as lifelikeness, might be specified quickly and automatically^16^. In fact, some researchers have reported rapid processing of single abstract items^17^. However, to date, no visual mechanism has been proposed to support the rapid extraction of abstract information from groups of objects in a visual scene. Here we explore whether ensemble coding supports rapid abstract impression formation.

Until now, ensemble perception has only been demonstrated to operate on basic visual dimensions^3^,^4^,^18^,^19^,^20^. However, even fleeting glimpses of visual environments engender a rich perceptual impression that cannot be easily explained by the summary statistics of basic visual features. For example, our impression of the liveliness and energy depicted in a photo of mounted animals at a natural history museum compared to a photo of those animals at the zoo is not well explained by ensemble perception of colour (similar), texture (similar) or even biological motion information (irrelevant in the case of static pictures). The context alone also does not give away the answer, as it depends on an interaction between the objects and context, among other factors. Museums, for example, can be more or less animate than zoos, depending on things like whether a busload of children is arriving. In this study, we tested whether summary statistical perception can precisely represent the vibrancy or lifelikenes*s* of random sets of stimuli. We find that observers perceive the average lifelikeness of crowds of objects, demonstrating that ensemble or summary statistical perception may underlie our perception of abstract visual experiences. This process could provide a link between summary statistical representations for basic features, objects and the kind of gist perception that observers report in their first glance impressions of visual scenes.

## Results

### Individual object lifelikeness ratings

**Figure 1:**
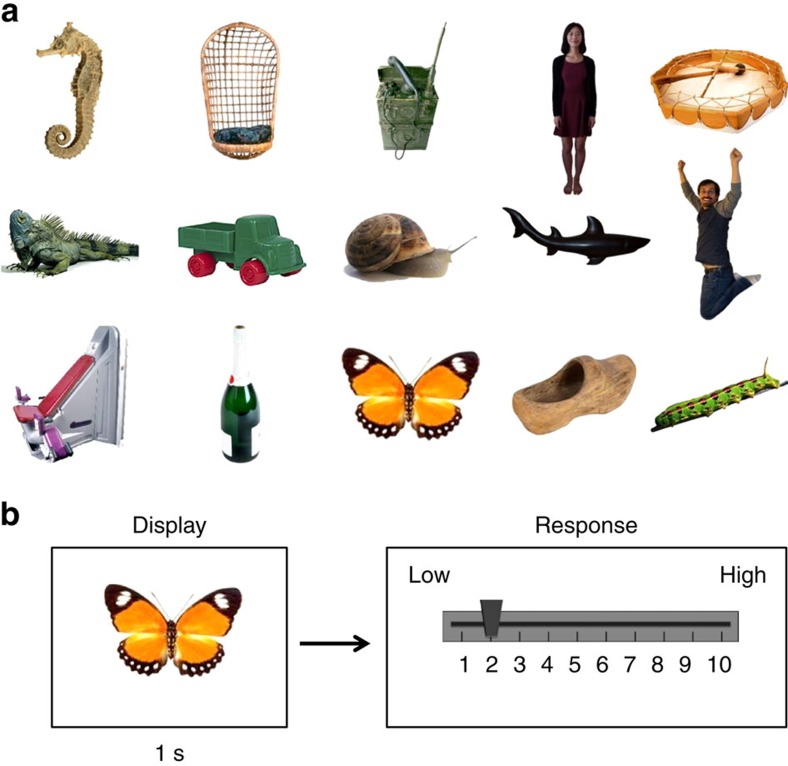
Stimuli and trial sequence in Experiment 1. (**a**) Example images illustrating the diversity of stimuli used in the experiment (out of 150 total). The images exhibit a wide variety of physical features, and some stimuli may be associated with both living and non-living attributes. Some images used in the actual experiments are replaced in these figures due to copyright. (**b**) Experiment 1 trial sequence. Participants viewed a random stimulus for 1 s and then rated it on a Likert scale of lifelikeness using a slider bar.

In the first experiment, 20 participants on Mechanical Turk rated the lifelikeness of 150 different static stimuli on a ten-point Likert scale (see Methods). The participants freely viewed each stimulus one time; no stimulus was repeated. In the instructions, lifelikeness (or animacy) was explicitly defined as ‘how relatively alive the item in the photograph appeared'. In each trial, a randomly selected stimulus (various objects, people, animals, insects, food, and so on) was displayed for 1 s. After the stimulus disappeared, a slider bar appeared with the words ‘Please rate the previously shown picture on a scale of 1–10', with one representing the lowest possible lifelikeness rating and ten representing the highest possible lifelikeness rating. Figure 1b depicts the trial sequence. Participants were not given a time limit to rate items, and they were not allowed to skip any items. After the response, the experiment advanced to the next trial. Participants completed 150 trials in total, and each participant viewed the stimuli in a randomly generated sequence.

We evaluated participants' consistency in rating lifelikeness by using an intra-class correlation coefficient test, or ICC^21^. Specifically, we used a mixed, two-way ICC model to measure consistency across the average ratings. The test yielded an ICC within the excellent range, ICC=0.976 (ref. 22). This ensures that observers agreed on the lifelikeness of objects, and suggests that lifelikeness was rated similarly across observers. After confirming inter-rater reliability, each stimulus was assigned the average value of the 20 participants' ratings for that particular item. The lifelikeness ratings ranged from 1.55 to 8.95.

### Ensemble coding lifelikeness in object groups

**Figure 2:**
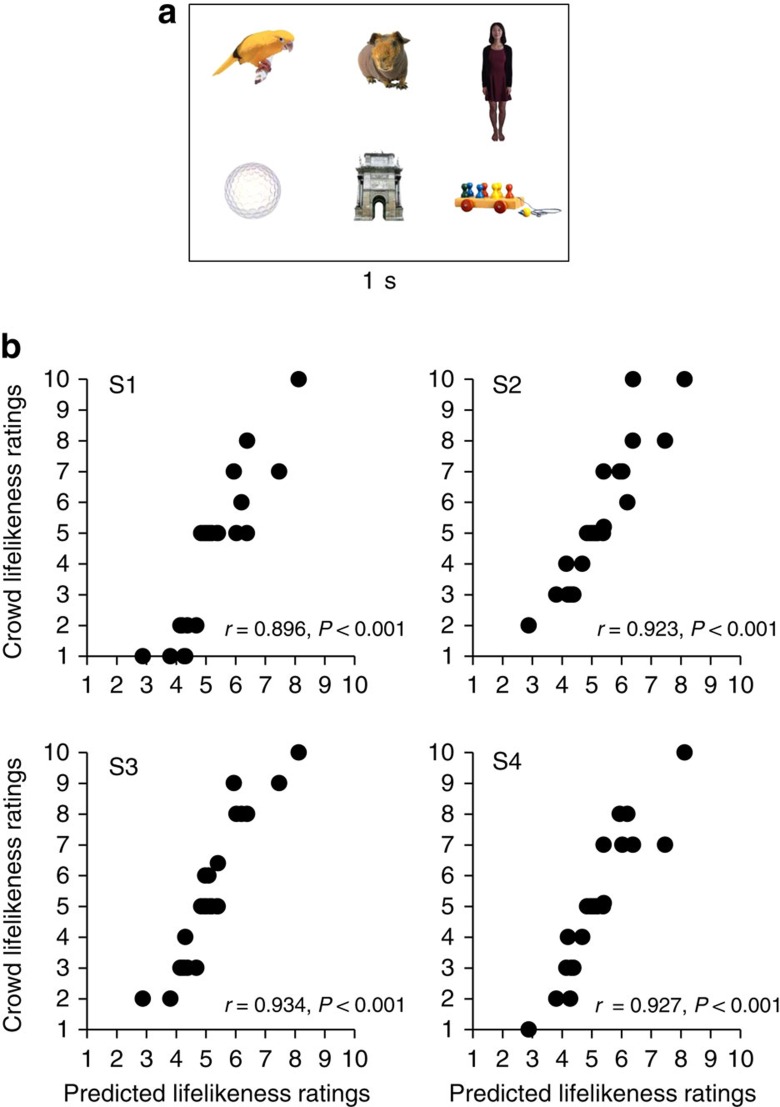
Experiment 2 stimuli and results. (**a**) In the whole set condition, a display of six stimuli was presented for 1 s. Participants then rated the average lifelikeness of the group of stimuli using a slider bar. (**b**) Experiment 2 results for four representative subjects. The observers' ratings (black circles) of crowd lifelikeness (*y* axis) were well predicted by the average of individual item ratings derived from an independent group of observers in Experiment 1 (*x* axis), using a Pearson correlation, *n*=25.

In the second experiment, we created groups of stimuli by randomly drawing six objects without replacement from the 150-item stimulus set that had been rated in the first experiment. This process yielded 25 groups containing 6 stimuli each (Fig. 2a; see Methods). Each of the 25 groups was assigned a single, independently determined predicted lifelikeness rating, calculated by averaging the ratings of the six objects comprising the group (from Experiment 1 data). From now on, the result of this calculation will be referred to as the ‘predicted' lifelikeness value of the group. The predicted lifelikeness ratings of the 25 groups were normally distributed around a mean of 5, with a range from 2.88 to 8.125.

Twenty new Mechanical Turk subjects participated in the second experiment. Their task was to judge the average lifelikeness of each group of six objects. The methods were identical to the first experiment, except participants freely viewed a group of 6 stimuli for 1 s, and were asked to ‘Please rate the average animacy of the previously shown group'. Participants were not given a time limit to rate the group, and were not allowed to skip any groups of stimuli. Importantly, stimuli were displayed in two ways. In the whole set condition, participants viewed the entire set of six stimuli. In the subset conditions, participants viewed subsets of the whole set. Specifically, in the subset conditions, either 1, 2 or 4 stimuli from the entire set were displayed to the participant. The subsets were randomly chosen from the whole set (see Methods). The whole set and subset conditions were randomly interleaved throughout the experiment, and every participant viewed the crowds in a randomly generated order. Each participant responded to 25 possible groups of objects at each of the four set size conditions (1, 2, 4 and 6), for a total of 100 trials.

If participants were able to extract the average lifelikeness from a group of visually distinct stimuli, we would expect the participants' ratings in the whole set condition to correlate with the predicted lifelikeness of the group (based on ratings of individual objects made by independent observers in the first experiment). For the set of 25 unique groups, we conducted a Pearson correlation test between participants' mean lifelikeness ratings of the groups in the second experiment and the predicted lifelikeness of the groups from the first experiment. Figure 2b shows four representative subjects' data in the whole set condition. This analysis was performed individually for all 20 participants. The averaged Fisher *z* value across participants, (*z*=1.08; *r*_*z*_′, *P*<0.001, *n*=20), suggests that participants were able to extract ensemble lifelikeness.

**Figure 3:**
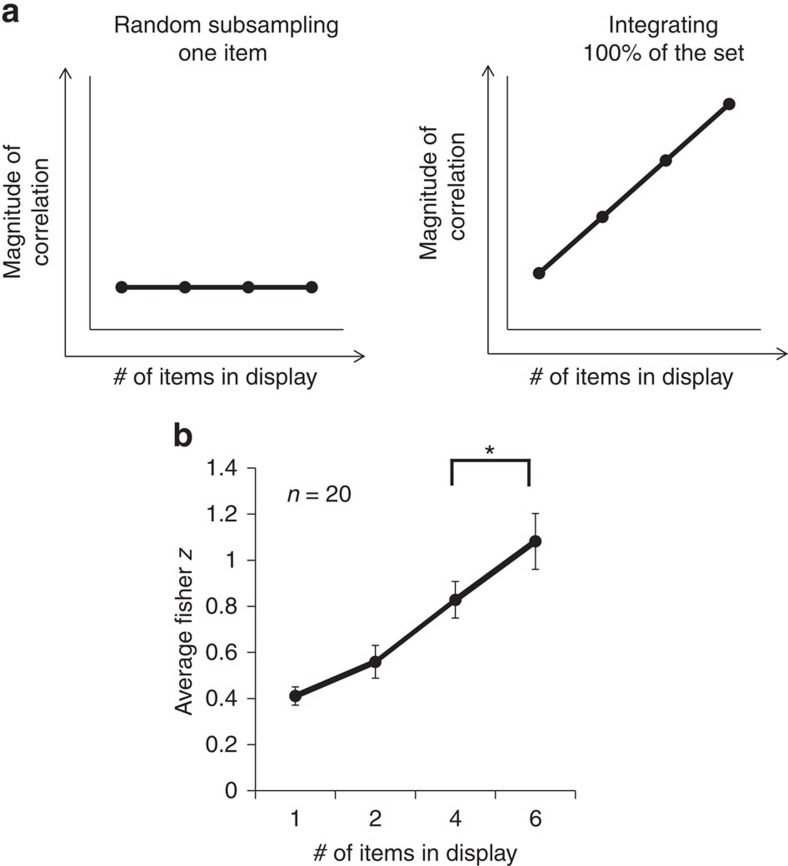
Hypothetical and empirical results for Experiment 2. (**a**) Hypothetical outcomes for the subset conditions, comparing participants' performance during random subsampling of one object or during ensemble coding (an extreme version of ensemble coding in which 100% of the objects in the set are integrated). Left: If the participant randomly samples lifelikeness information from just one item in the set, the magnitude of the correlation should remain relatively constant, even when more information becomes available because the participant does not use the new information. Right: In contrast, if the participant integrates lifelikeness information from every item as it becomes available to them, the correlation between participants' lifelikeness ratings and the predicted lifelikeness ratings of the crowd should increase as more information (more items) becomes available. (**b**) Experiment 2 Results. The Fisher *z* scores increase as the number of items displayed increases. This pattern indicates that participants integrated the available information and did not use a random subsampling technique to accomplish the task. Error bars represent s.e.m.

While the results indicate that participants perceived ensemble lifelikeness, it is critical to determine whether the observers actually integrated lifelikeness information from multiple items in the group or merely randomly sampled a single item from the group. The subset conditions allow us to simulate what participants' responses in the whole set condition would look like if they randomly selected a single stimulus from the group of pictures or randomly sampled small subsets from the group of pictures^20^,^23^,^24^. The subset conditions make an important prediction: If participants engaged in ensemble coding, their ratings of lifelikeness in the whole set condition would be more highly correlated with the predicted lifelikeness value of the entire group compared to the subset conditions. The correlations should increase monotonically as set size increases. We will refer to this outcome as the ‘subset effect'. The logic is as follows: When lifelikeness information about the whole set of stimuli is present, observers will use it. By the same logic, if participants integrate the presented objects into an ensemble, correlations will decrease with smaller subset sizes. This is because the randomly selected subsets will not always be representative of the overall lifelikeness of the group. Importantly, we will only observe this outcome if the participants actually integrate lifelikeness information from multiple objects. Conversely, if participants based their lifelikeness judgement on a single randomly sampled object, their performance (that is, correlation between observers' crowd ratings of lifelikeness and the predicted ratings) across the subset conditions would not show an improvement as more information became available (Fig. 3a, left). Instead, their performance would asymptote at a small subset size, indicating that they were using a subsampling strategy to accomplish the task. Figure 3a illustrates the two expected patterns of performance for random subsampling and for integrating 100 percent of the items, an extreme form of ensemble coding.

### Subset condition control results

For each subject and subset condition, we calculated a Fisher *z* (correlation) between participants' ratings of set lifelikeness and the predicted lifelikeness of the whole set (from Experiment 1). The average Fisher *z* scores across subset conditions were well fit by a linear regression, *r*^2^=0.998, *P*<0.001, *n*=4, illustrating that participants exhibited increasing correlations as set sizes became larger (Fig. 3b).

These results confirm that subjects were not engaging in a strategy of sampling one object or relying on the most extreme object to accomplish the lifelikeness rating task. Instead, these results indicate that participants used most of the available information—a hallmark of ensemble coding. Additionally, we conducted a permutation test on the Fisher-transformed data to compare participants' correlation values between the four-object subset and the whole set. Participants exhibited a higher correlation in the whole set than the four-object subset, *P*<0.001, *n*=20. This indicates that participants integrated information from more than four objects in the set.

### Fast ensemble lifelikeness perception

**Figure 4:**
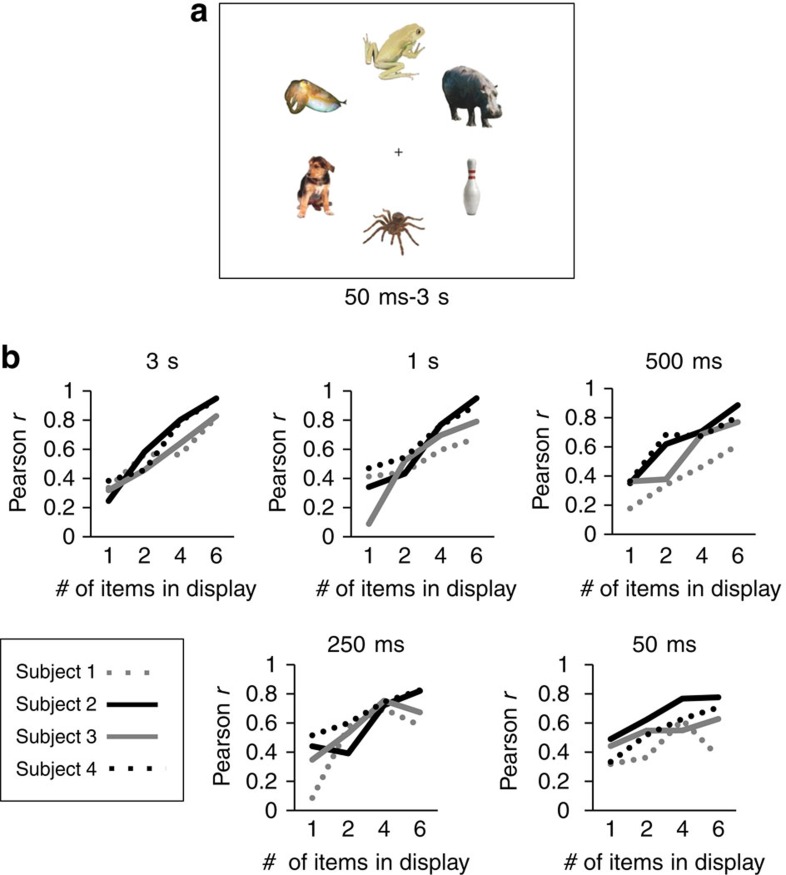
Ensemble coding lifelikeness at different display durations. (**a**) Example display in Experiment 3. Participants viewed groups of up to six stimuli presented for 50 ms to 3 s, then rated the average lifelikeness of the group. (**b**) Participants consistently integrated multiple items across display durations.

The data from the second experiment indicated that participants perceive ensemble lifelikeness in sets of objects viewed for 1 s. An open question, in Experiment 3, is how quickly ensemble lifelikeness perception can operate. To test this, and to confirm that the results of the second experiment extend to a laboratory setting, we replicated the second experiment at five different exposure durations with experienced psychophysical observers (see Methods). Subsets of stimuli (as in the second experiment) were presented for durations of 50 ms, 250 ms, 500 ms, 1 s or 3 s. In each trial, participants foveated on a fixation cross, and viewed up to six stimuli, which were displayed isoeccentrically around the fixation cross (see Fig. 4a, also see Methods). The different exposure durations and subset conditions (1, 2, 4, 6 stimuli per set) were randomly interleaved (52 trials each), totalling 1040 trials per participant.

We observed highly robust correlations between participants' estimates of ensemble lifelikeness and the predicted lifelikeness values of the crowds for all exposure durations (Fig. 4b). The participant's Fisher *z* scores in each exposure duration condition were well fit by a linear regression (lowest *r*^2^ was 0.37, in the 50 ms set duration condition, *P*=0.013, *n*=16) illustrating the increasing correlations for larger set sizes. To confirm that ensemble lifelikeness perception does not rely on visual or monitor persistence, we conducted two control experiments that replicated the results of this third experiment with backward masking of the briefest displays (Experiments 8 and 9, Supplementary Fig. 1, Supplementary Table 2). These results reinforce the results of Experiment 2 (data from Mechanical Turk) within a controlled laboratory setting, indicating that it is possible to formulate an ensemble percept of visually distinct items. These results also provide a hint that participants integrated multiple stimuli into their estimates of ensemble lifelikeness, even for briefly presented sets. Although this is intriguing evidence that ensemble lifelikeness perception may be a fast process, there remain unanswered questions. First, how many stimuli are integrated in a brief glance? And, second, do subjects rely on explicit memory of the stimuli? To address these questions, we conducted a follow-up experiment.

### Limited explicit memory for set members

The third experiment indicated that observers perceive ensemble lifelikeness even for briefly presented sets. Although this could indicate a fast perceptual process, an alternative is that observers recall the items in the set and use this memory to make their judgement of lifelikeness. It is therefore necessary, in Experiment 4, to determine whether group lifelikeness perception occurs without explicit memory for individual items in the group. To address this, we needed a more precise estimate of how many stimuli observers integrated into their ensemble lifelikeness percept, and we needed to measure memory capacity for the objects in the sets. To this end, we presented sets of objects in two conditions, measured in separate runs.

In the first condition, we replicated the third experiment, presenting sets of 1, 2, 4 or 6 objects for 250 ms. Like in Experiment 3, we measured correlations between participants' estimates of ensemble lifelikeness and the predicted lifelikeness values of the crowds from independent observers. In the second condition, the sets of stimuli were similar but the observers performed a membership identity task. In each trial, subjects viewed six stimuli displayed isoeccentrically from a central fixation cross for 250 ms. After the set disappeared, two items appeared on the screen. One item was a member of the previous set, and one item was a lure (drawn from the full set of 150 images). In a two-alternative forced choice task, participants chose which stimulus was a member of the previous set by pressing one of two keys on a keyboard. Memory capacity was estimated from the proportion correct in this task (see Methods). Observers participated in 100 trials in each condition.

**Figure 5:**
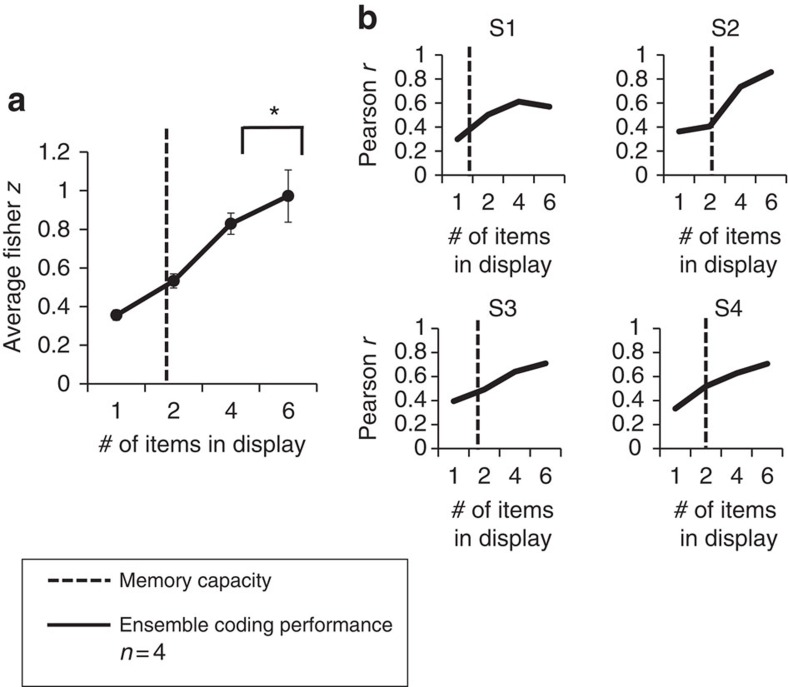
Limited explicit memory of all items. (**a**) Observers perceived ensemble lifelikeness in the 250 ms condition in Experiments 3 and 4 by integrating five or more objects (indicated by the significant difference in performance when four and six items are viewed). Separate memory capacity testing revealed that participants only recalled less than two items per display, on average (black dashed line). Error bars represent s.e.m. (**b**) Individual subject data.

Results for the first condition replicated and extended the third experiment. The average Fisher *z* score was 0.972, *r*_*z*_′, *P*<0.001, *n*=4. We also fit a linear model to participant's Fisher *z* scores (collapsing across the 52 trials in Experiment 3 and 100 trials in Experiment 4) during the 250 ms exposure condition and found a significant positive linear trend (linear model *r*^2^=0.75, *P*<0.001, *n*=16). On average, participants integrated up to six stimuli in their estimates of ensemble lifelikeness (see Fig. 5a). A permutation test comparing participants' Fisher *z* values between the four-object subset and the whole (six object) set revealed a significant difference, *P*=0.02, *n*=4, indicating that observers integrated up to six objects in their estimates of ensemble lifelikeness. A regression analysis^25^ complemented these findings, and indicated that participants integrated all display items into their ensemble percept (Supplementary Tables 1 and 2).

The second condition measured memory capacity for objects in the briefly presented sets. On average, participants exhibited an effective memory capacity of 1.89 items, when sets of six stimuli were presented (vertical dashed lines in Fig. 5a,b; see Methods). Taken together, the results demonstrate that perception of group lifelikeness cannot be based on explicit memory alone, or on a cognitive calculation that relies on the explicit memory of each item. Ensemble lifelikeness can be perceived even when the individual set members are lost or forgotten.

### Perceiving group lifelikeness over time

The first four experiments required participants to extract an ensemble percept from items arranged in a spatial array, demonstrating that ensemble lifelikeness perception involves some degree of spatial integration. Because of eye and object motion, however, we often encounter objects and crowds in dynamic situations. In Experiment 5, we tested whether observers can perceive ensemble lifelikeness in a temporal sequence^26^,^27^.

**Figure 6:**
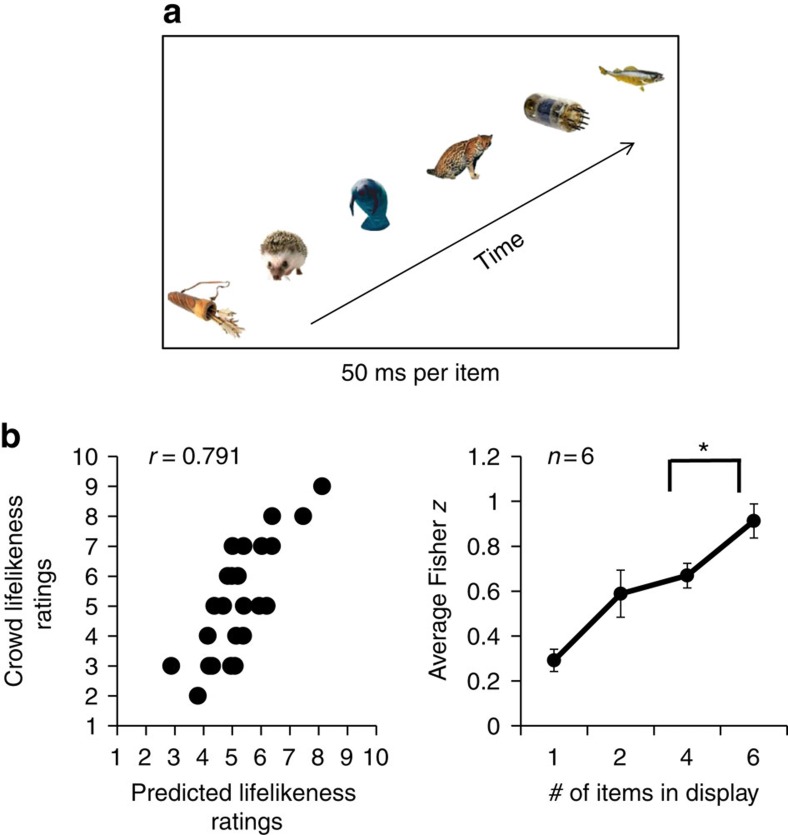
Example stimuli and results for Experiment 5. (**a**) In the whole set condition, participants viewed groups of six stimuli, sequentially presented for 50 ms each. Participants then rated the average lifelikeness of the previously seen group. (**b**) Experiment 5 results. Left: Crowd lifelikeness of temporally presented groups is well predicted by the average of individual item ratings. This graph depicts the ratings (black circles) of one representative participant who judged 25 unique temporal crowds in the whole set condition. There is a high correlation between the ratings of average crowd lifelikeness (*y* axis) and predicted ratings of the crowds generated from single-item ratings (*x* axis), *n*=25. Right: Average results for six observers. The *x* axis represents the number of items displayed in the set. The *y* axis represents the magnitude of the Fisher *z* score. The Fisher *z* score in the whole set condition indicates that participants were able to perceive ensemble lifelikeness in sequentially presented groups of items. Moreover, Fisher *z* scores increase as the number of items displayed increases. This pattern rules out the possibility that participants engaged in random subsampling to accomplish the task. Error bars represent s.e.m.

In this fifth experiment, six participants in the laboratory viewed the same sets of stimuli viewed by Mechanical Turk subjects in the second experiment. However, as shown in Fig. 6a, these items were displayed sequentially over time, rather than simultaneously over space. After the items disappeared, a blank screen appeared, during which participants rated average the lifelikeness of the set. Participants used buttons labelled 1–10 on the computer keyboard, where 1 represented the lowest lifelikeness rating and 10 represented the highest lifelikeness rating. There were four conditions, corresponding to four different set sizes (1, 2, 4 or 6 objects per set). The whole set condition contained six items displayed foveally with a spatial jitter (see Methods) for 50 ms each. In the subset conditions, participants viewed each item for a longer duration to equalize the total visible stimulus duration (see Methods). Note that, in many ways, increasing the exposure duration for the items in the smaller subsets actually works against the subset effect (Fig. 3a right panel), as the longer exposure could facilitate recognition or memory. Therefore, performance might be expected to increase (or stay constant) in the small subset conditions. Despite this, if participants truly integrate lifelikeness information from most of the items displayed, we will still observe the predicted subset effect (Fig. 3a right panel) because the subset is not truly representative of the average of the whole crowd. Each participant completed 100 trials in total (25 possible groups of objects × 4 set sizes).

If observers are able to extract the average lifelikeness from a group of sequential stimuli, we would expect lifelikeness ratings in the whole set condition in this experiment to correlate with the predicted lifelikeness of the crowd based on the ratings of individual objects made by independent observers (from Experiment 1). Once again, we conducted a bivariate correlation test between participants' lifelikeness ratings of the temporal crowds presented in Experiment 5 and the predicted lifelikeness values. Figure 6b left panel shows the results from one representative participant, Pearson *r*=0.791, *P*<0.001. We transformed the Pearson correlation coefficients to Fisher *z* scores and averaged across participants (Fisher *z*=0.913, *r*_*z*′_=0.722, *P*<0.001, *n*=6). A linear regression of average group performance versus set size demonstrated that participants' performance became more correlated as set size increased, *r*^2^=0.905, *P*=0.048, *n*=4 (Fig. 6b, right panel). A permutation test revealed a significant difference between the four-item subset and the whole set, *P*<0.001, *n*=6, with participants exhibiting a higher correlation in the whole set versus the four-item subset. This indicates that participants integrated five or more items into their ensemble percept. It also confirms that participants were not using a random subsampling strategy, and reveals that participants can ensemble code lifelikeness temporally as well as spatially.

### Visual short-term memory control

A common question in ensemble coding literature is whether attention or awareness of single objects is necessary to formulate an ensemble percept. Many previous experiments indicate that participants are able to formulate a remarkably precise ensemble percept of low-level stimuli (simple shapes, orientation or motion) or high-level stimuli (face, biological motion), even when performance at membership identity tasks is relatively poor^3^,^28^,^29^,^30^. Ensemble animacy perception appears to follow a similar pattern: Experiment 4 found that observers had memory capacity of less than two effective objects, and yet they integrated more than five into their ensemble percept. However, that experiment employed spatial arrays. An open question is whether the observers lack information about the individual objects in the temporal array (Experiment 5). In the sixth experiment, we therefore investigated whether participants were able to formulate an ensemble percept of a temporal sequence of objects without specific memory of every item in the group.

In the sixth experiment, we also increased the set size to 12 items. This allowed us to ensure that the results remained robust with large set sizes. In this experiment, we pseudo-randomly generated crowds (see Methods) for each different participant.

Five participants in the laboratory viewed temporally presented sets of 12 stimuli. Stimuli were presented foveally with a spatial jitter (see Methods). The subset conditions consisted of 1, 2, 4 and 8-item subsets. Each stimulus was shown for 50 ms, with a 50 ms interstimulus interval (ISI) in all set sizes. Whole (12-item) set and subset conditions were randomly interleaved. Observers participated in a total of 510 trials, with 102 trials per set size condition.

**Figure 7:**
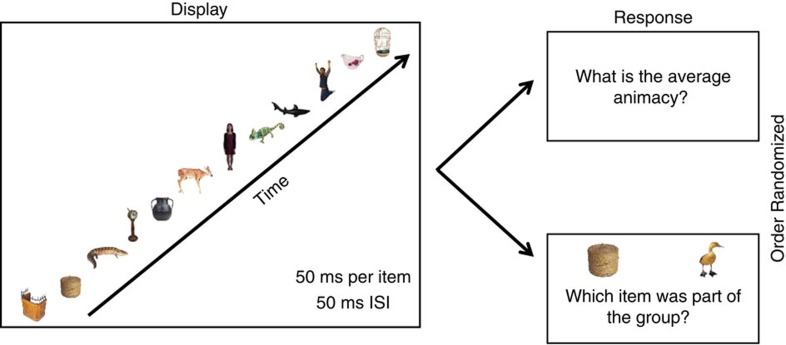
Trial sequence for the whole set condition. First, participants viewed 12 random stimuli displayed sequentially for 50 ms per item, with a 50 ms ISI. Next, participants viewed a two-alternative forced choice membership identity task *and* participants were asked to rate the average lifelikeness of the group. Participants were not given a time limit to complete both tasks. The order of the two tasks was randomized throughout the experiment, so that sometimes participants performed the lifelikeness rating first and sometimes participants performed the membership identity task first.

A layout of the experimental trial sequence is depicted in Fig. 7. First, participants viewed the temporal display of stimuli. Participants then performed two randomly ordered tasks. In one task, they rated the average lifelikeness of the group. In the other task, participants performed a 2AFC membership identity task (identical to Experiment 4). In the 2AFC task, two stimuli were shown on the screen side by side. One of the stimuli was a lure; the other stimulus was a randomly selected item from the previously seen set. Participants chose which object was a member of the set (see Methods). The order of the tasks was randomized. That is, in some trials, the participants performed the membership identity task directly after viewing the crowd, and then rated the lifelikeness of the crowd. In other trials, participants rated the lifelikeness of the crowd directly after viewing the crowd, and then performed the membership identity task. Participants were not given a time limit to perform either task (membership identity and lifelikeness ratings). As soon as the participants entered their response, the other task appeared.

**Figure 8:**
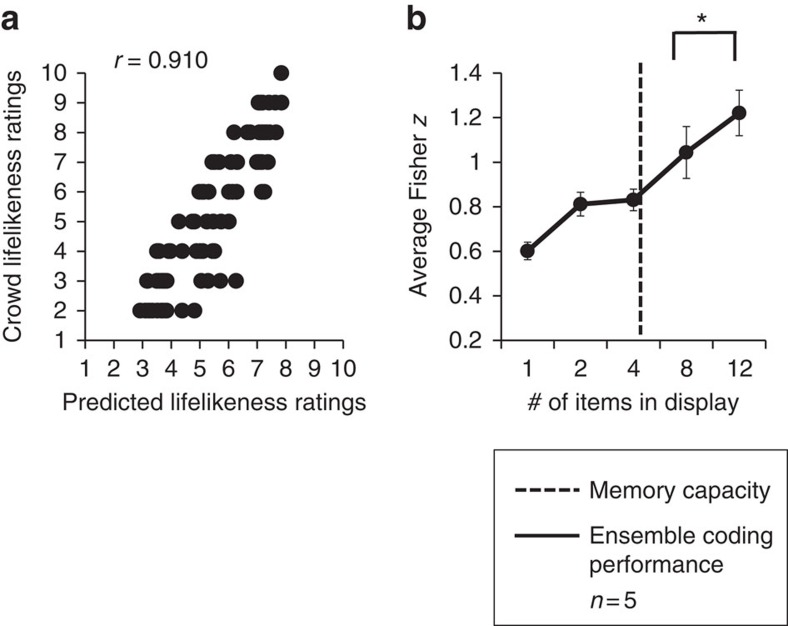
Results of Experiment 6. (**a**) Crowd lifelikeness of large groups is well predicted by the average of individual item ratings from independent observers. This graph depicts the ratings (black circles) of one representative participant judging 102 unique temporal groups. There is a high Pearson correlation, *r*=0.910 between the ratings of average crowd lifelikeness (*y* axis) and predicted ratings of the groups generated from single-item ratings (*x* axis). (**b**) Averaged data for five observers. The *x* axis represents the number of items displayed in the set. The *y* axis represents the magnitude of the Fisher *z* score. The Fisher *z* score in the whole set condition indicates that participants are able to perceive ensemble lifelikeness even in large groups of items (in this case 12 items). The Fisher *z* scores increase as the number of items displayed increases. This pattern rules out the possibility that participants engaged in random subsampling techniques to accomplish the task. Error bars represent s.e.m. Finally, the dashed line represents the memory capacity limit in this experiment. Participants remembered on average five items in each display. However, their ensemble coding performance continued to significantly increase between 8 and 12 items, indicating that ensemble coding performance is not solely dependent on explicitly remembered items.

As in the previous experiments, we correlated participants' lifelikeness ratings of the whole set with the predicted lifelikeness ratings based on an average of the individual objects within the set (as determined by independent observers in the first experiment). In this particular experiment, the whole set was twice the size of whole sets in previous experiments (that is, 12 versus 6 items). We still observed a robust correlation between the participants' lifelikeness ratings and the predicted lifelikeness of the group (Fig. 8b; Fisher *z*=1.22, *r*_*z′*_, *P*<0.001, *n*=5). A representative participant's data are shown in Fig. 8a.

We examined participants' performance in the different set sizes by examining participants' average Fisher *z* scores in the subset conditions. The average Fisher *z* values across the different set sizes were well fit by a linear model, suggesting that performance improved as set size increased, *r*^2^=0.942, *P*<0.001, *n*=4 (Fig. 8b). Additionally, a permutation test comparing Fisher *z* values between the eight-item subset and the whole (12-item) set indicated a significant difference, *P*=0.002, *n*=5, with participants exhibiting a higher correlation in the whole set versus the eight-item subset. This indicates that participants integrated more than eight items into their ensemble percept of lifelikeness.

We analysed the number of items participants remembered in each trial by calculating a measure of memory capacity (see Methods). Participants' memory improved in the smaller subsets, but plateaued for the larger set sizes. Participants remembered approximately 1 item in the 1-item display, 2 items in the 2-item display, 3 items in the 4-item display, and 5 items in both the 8-item set and the 12-item display (average effective memory capacity (MC) in the 1-item set=0.98 (SEM=0.004); MC in the 2-item set=1.92 (SEM=0.005); MC in the 4-item set=2.96 (SEM=0.016); MC in the 8-item set=4.64 (SEM=0.009), MC in the 12-item set=4.8 (SEM=0.008)). Even though participants remembered approximately 5 items in both the 8-item display and the 12-item display, their ensemble coding performance continued to substantially increase between set sizes 8 and 12. This indicates ensemble coding without explicit memory for individual items in the temporal display. This pattern of results closely mirrors the results in Experiment 4, where explicit memory for individual objects in a spatial array of objects was not necessary for perception of ensemble lifelikeness.

### Binary versus graded representations

Experiments 2, 3 and 5 suggested that participants perceived ensemble lifelikeness over space and over time. Experiments 4 and 6 replicate these findings, and suggest that explicit memory of individual objects was not required to form ensemble representations of lifelikeness. However, it remains unknown how participants extract the average lifelikeness of the group. One potential strategy is that participants perceive lifelikeness as categorical and binary (either alive or not) and then average the items (we refer to this as the ‘binary' strategy). Alternatively, observers might perceive graded or relatively nuanced differences in the lifelikeness of individual items to achieve a precise ensemble percept of the group as a whole. We refer to this strategy as a ‘graded averaging strategy'. Both perceptual strategies could result in an ensemble representation, and both strategies may be relied upon under different circumstances, but they make different predictions.

**Figure 9:**
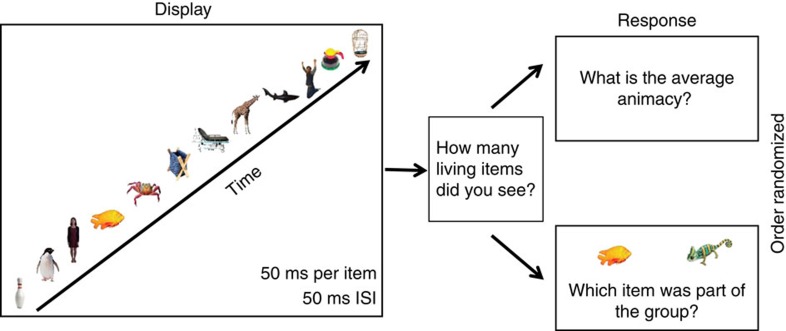
Trial sequence in Experiment 7. Participants were instructed to count the number of living items in the display. They viewed 12 random stimuli displayed sequentially for 50 ms per item with a 50 ms ISI. Then, participants verbally reported their count to the experimenter. Afterward, the participants performed both the 2AFC membership identity task and rated the average lifelikeness of the group.

To investigate this, in Experiment 7, we used a design similar to the sixth experiment except that participants were instructed to count either the number of living or non-living items in each group (counterbalanced across participants). The trial procedure was as follows: Participants viewed a temporally displayed crowd, then after each display, participants verbally reported their count to the experimenter. After their verbal report, participants rated the ensemble lifelikeness of the group and performed the membership identity judgement in a randomized order, similar to Experiment 6 (see Fig. 9 for task layout). The counting task allowed us to create a binary averaging model: a simulation of what participants' performance would look like if they were simply assigning binary values (for example, 1's and 10's, or 4's and 6's, and so on) to the non-living and living items respectively, and then averaging these numbers. Observers participated in 24 trials total.

Both strategies (binary averaging and graded averaging) will, unsurprisingly, produce a similar pattern of results. However, if participants extract graded information, we should still observe a slight decrement in performance in the binary averaging simulation compared to their actual ensemble coding judgement. This is exactly what we found. We fit a linear regression through the origin of participants' ensemble coding performance and the performance predicted from the binary averaging model. Participants' graded averaging was a better fit (graded averaging *r*^2^=0.941, binary averaging *r*^2^=0.777). To ensure that participants' ensemble rating judgements were not biased by the counting task, we also compared the same binary averaging simulation for each participant to their averaging performance in a separate task. This separate measure of ensemble coding performance was identical to Experiment 7 (excluding the counting task), and the result was similar (graded averaging *r*^2^=0.934, binary averaging *r*^2^=0.777). Thus, our results suggest that participants did not solely rely on a binary ensemble coding strategy when viewing sets of sequentially presented stimuli. We also tested whether observers use graded versus binary averaging when judging animacy in spatial arrays of stimuli. We found that participants did not rely on a binary ensemble coding strategy (Experiment 10, Supplementary Fig. 2). Additional control experiments ensured that participants did not merely rely on low-level features or image statistics to determine lifelikeness (Experiments 11 and 12; Supplementary Figs 3 and 4). Instead, participants relied on configural information^31^,^32^,^33^ to assess the liveliness of a group.

## Discussion

Our results indicate that observers can perceive the average lifelikeness of groups of objects in a fraction of a second. This is the first evidence that ensemble coding may contribute to our first glance or gist impression of abstract attributes like animacy or liveliness. Our results show that gist impressions of visual scenes are rich: they encompass more than a sparse statistical summary of concrete physical dimensions. Until now, it was unknown whether individuals could extract an ensemble code from perceptual impressions that were not immediately specified by the visual features in the image. Our experiments demonstrate that individuals can extract ensemble percepts about abstract visual interpretations, suggesting that lifelikeness is an explicitly coded perceptual dimension. Moreover, these representations are remarkably consistent across observers, suggesting that lifelikeness is a shared visual percept. Our results provide a link between summary statistical representation of basic visual features, and the vibrant, complex perceptions that observers report experiencing in their first impressions of a visual scene.

Our findings reveal that ensemble perception of lifelikeness is achieved extremely rapidly. While previous work has shown that observers categorize stimuli in a brief time period (for example, animal or non- animal^34^,^35^), our study shows that observers can perceive relative lifelikeness (that is, whether one stimulus is more life-like than another) on a similarly rapid timescale for groups as well. These results parallel the rapid time scale reported in previous ensemble coding experiments using stimuli with explicit physical dimensions^24^,^26^, highlighting the remarkable efficiency of ensemble representations that support abstract visual impressions.

Our findings suggest that lifelikeness is an explicitly coded perceptual dimension that is continuous as opposed to dichotomous. One prior study has investigated whether animacy is a strictly dichotomous representation, or whether animacy is represented as a continuum^36^. While this prior study focused on single repeated stimuli shown for longer exposure durations, our findings extend this question to groups of heterogeneous objects that were briefly presented. Our participants extracted a graded ensemble percept of group lifelikeness. Because of the rapid timescale, the judgements of lifelikeness in our experiment would not allow for cognitive reasoning or social processes. Consistent with this, explicit memory of the objects in the sets was not sufficient to account for the number of objects integrated into the ensemble percept. Our results suggest that graded representations of object and crowd lifelikeness emerge as a basic, shared visual percept, available during rudimentary and rapid visual analysis of scenes.

Animacy, as a general construct and topic of cognition research, is extremely complex. Numerous contextual, cognitive and social mechanisms come into play when determining whether an object exhibits animate qualities. Specifically, when making judgements about animacy, theory of mind^37^,^38^,^39^, contextual cues^40^,^41^ and cognitive strategies^42^ contribute significantly to animacy evaluations. These complexities help explain why there are relatively few agreed-upon operational definitions of animacy or lifelikeness.

In contrast to the ambiguity of the terms animacy or lifelikeness, our results show that the ensemble perception of lifelikeness in groups of static objects was surprisingly consistent across observers. When stimuli were presented for brief durations, observers reached a remarkable consensus on the average lifelikeness—even regarding objects that exhibit seemingly ambiguous qualities. This consistency suggests that a similar percept of lifelikeness is commonly available to observers who glance at a scene. Numerous cognitive and social mechanisms may come online later, and observers may refine their percepts of lifelikeness when given longer periods to evaluate items and context. However, in a first-glance impression of the environment, observers share a relatively unified, consistent percept of lifelikeness.

## Methods

### Participants

In total we tested 68 healthy participants with normal or corrected-to-normal vision. In Experiment 1, we tested 20 participants on Amazon Mechanical Turk (mean age=34.89, SD=13.75; 12 males, 8 females). In Experiment 2, we tested 20 new participants on Amazon Mechanical Turk (mean age=40.9, SD=14.164; 6 males, 14 females). In Experiments 3 and 4, we tested four experienced psychophysical observers (mean age=27.75 SD=5.31; 2 males, 2 females). In Experiment 5, we tested six experienced psychophysical observers, including one author (mean age=26.833, SD=5.193; 3 males, 3 females). In Experiment 6, we tested five experienced psychophysical observers, including one author (mean age=24, SD=5.148; 1 male, 4 females). In Experiment 7, we tested eight observers in the laboratory (mean age=26.625, SD=6.435; 5 females, 3 males). In Experiment 8, we tested three experienced psychophysical observers (mean age=27, SD=6.245; 2 females, 1 male). In Experiment 9, we tested two experienced psychophysical observers (mean age=25, SD=7.071; 1 male, 1 female). In Experiment 10, we tested four experienced psychophysical observers (mean age=27.75 SD=5.31; 2 males, 2 females). In Experiment 11, we tested three experienced psychophysical observers (mean age=27.75 SD=5.31; 2 females, 1 male). In Experiment 12, we tested 14 participants from Amazon Mechanical Turk (mean age=35.83 SD=7.49; 3 females, 3 males, for those who reported demographic information). Amazon Mechanical Turk observers who failed to complete all of the experimental trials were automatically excluded from the experiment and any subsequent data analysis. Some observers who were tested in the laboratory participated in multiple experiments. All participants, with the exception of the one author, were naïve as to the purpose of the experiment. Each participant provided informed consent in accordance with the IRB guidelines of the University of California at Berkeley.

### Stimuli

We used the stimuli from the Massive Visual Memory Stimulus database^43^. This stimulus set contains coloured photos of diverse objects including electronics, household items, food, plants, people, animals, insects, vehicles, furniture and many other items on a white background. As the database may be heavily biased towards living or non-living objects, we first very roughly balanced or flattened the distribution of living and non-living stimuli. To accomplish this, the database was coarsely divided into living and non-living stimuli by one observer. This does not reflect an objective measure or a reference baseline of lifelikeness, but was done to simply increase the likelihood that participants potentially viewed a broad distribution of items. Participants were unaware of this step or that the experimental stimuli were approximately balanced in this way. Of course, individual observers might still perceive the distribution of stimuli as heavily biased towards or away from living or non-living categories. From these divided stimuli, we randomly chose 75 nominally living and 75 nominally non-living items. Figure 1a shows a representative subset of the images used in the experiment. The stimuli were presented either using Qualtrics (© 2009) for online participants or Psychophysics Toolbox^44^,^45^,^46^ in Matlab for laboratory experiments. Participants on Amazon Mechanical Turk were asked to place their personal computer monitor in a centred position in front of them, and were asked to maintain a clear, unobstructed view of the pictures and sit an arm's length away from the computer screen.

Participants in the laboratory viewed stimuli on a 68.6 cm iMac LCD monitor with resolution of 2,560 × 1,440 and 60 Hz refresh rate. Participants sat with the screen positioned centrally in front of them at a viewing distance of 60 cm. Each stimulus was presented in a white box, with boundaries subtending 4.61° × 4.61° of visual angle. In Experiment 2, each group of images was arranged on a grid with three stimuli on the top row and three stimuli on the bottom row within a 436 × 654 pixel grid. The location of each stimulus was randomly determined within the grid. The size of each stimulus was 218 × 218 pixels, and participants were allowed to freely view stimuli. In Experiments 3, 4, 8, 9 and 10, participants were instructed to foveate on a fixation cross. The visual angle between the fixation cross and stimuli was 6.98°. In Experiments 5–7 and 11, each stimulus was presented sequentially at the centre of the screen, with a spatial jitter of up to 4.14° on the vertical axis, 3.30° on the horizontal axis. Across all the above-mentioned experiments, the maximum and minimum luminance in the pictures was 327.5 and 1.38 cd/m^2^ respectively. The maximum Michelson contrast was 0.992. The visual angle and Michelson contrast in the remaining experiments (Experiments 1, 2 and 12) was not measurable, as these experiments were conducted on Mechanical Turk.

The stimuli were randomized in the following manner to create crowds of stimuli: In Experiments 2 and 5 we randomly drew from the entire stimulus array (150 items) without replacement to generate 25 displays of 6 stimuli. This random method yielded a broad range of predicted lifelikeness values for the crowds, from 2.88 to 8.125. Each participant viewed the 25 crowds in a random order. In Experiments 3, 4, 8 and 9 the crowds of objects were randomly generated for each participant on each trial. In Experiments 6, 7 and 11, we pseudo-randomly drew from the original stimulus set (150 items) to generate crowds of 12 stimuli. The one constraint was that one-third of the randomly drawn groups were below a predicted mean lifelikeness of 4, one-third of the groups had a predicted lifelikeness mean of 4–7, and one-third of the groups had a predicted mean lifelikeness above 7. This method ensured a similarly broad range of predicted lifelikeness ratings for the crowds, despite the fact that we incorporated twice the number of items for each display. In Experiment 10, we pseudo-randomly drew from the entire stimulus set, with the constraint that both sides of the display contained the same number of animate items, either 2 animate items and 1 inanimate item on each side of the display, or 2 inanimate items and 1 animate item on each side. In Experiment 12, we randomly generated 5 sets of stimuli, each containing 100 crowds of 6 randomly selected objects and 100 crowds of scrambled objects (Supplementary Fig. 4). Amazon Mechanical Turk participants were randomly assigned to one of the five stimulus sets.

### Procedure

Experiments 2–12 incorporated the following general trial layout: First, participants viewed a group of stimuli. Next, participants were asked to rate the average lifelikeness of the groups of stimuli. The different experiments included different display durations, number of stimuli and response methods. Specifically, in Experiment 2, six stimuli were shown for 1 s. In Experiment 3, six stimuli were shown for 50 ms, 250 ms, 500 ms, 1 s, and 3 s in interleaved trials. In Experiments 4, 8 and 10, six stimuli were shown for 250 ms. In Experiment 5, six stimuli were shown sequentially for 50 ms each in the whole set condition. To equalize total duration, the subset condition stimuli were shown for longer (83 ms per item in the 4-item subset condition, 150 ms per item in the 2-item subset condition, 300 ms per item in the 1-item subset condition). In Experiments 6 and 7, twelve, eight, four, two and one, stimuli were shown sequentially for 50 ms in interleaved trials. In Experiment 11, twelve stimuli were shown sequentially for 50 ms in the whole set condition. To equalize total duration, the subset condition stimuli were shown for longer (100 ms per item in the 8-item subset condition, 250 ms per item in the 4-item subset condition, 550 ms per item in the 2-item subset condition, 1,150 ms per item in the 1-item subset condition). Across all sequentially presented displays (Experiments 5, 6, 7 and 11), the ISI was 50 ms.

In all the experiments, after the display disappeared, participants were required to rate the average lifelikeness of the stimuli. In Experiments 1, 2 and 12, participants used a slider bar to rate the lifelikeness of the stimuli. The slider bar appeared after the display disappeared, and allowed the participants to rate the animacy of the display. Each end of the slider bar also had written cues reminding the participant that 1 represented the lowest and 10 represented the highest possible animacy or lifelikeness. The scale was integer based (that is, decimal ratings were not available to the participant). In Experiments 3, 4, 5, 6, 7, 8, 9, and 11, participants used keyboard buttons to rate the lifelikeness of the stimuli, with 1 representing the lowest lifelikeness and 10 representing the highest lifelikeness. In Experiment 10, participants also used keyboard buttons to indicate whether the crowd with the highest average lifelikeness was displayed on the left or right side of the screen. Across all experiments, participants were not given a time limit to make their response.

In addition to asking participants to rate the average lifelikeness of stimuli, the memory experiments (Experiments 4 and 6) also included a memory test during the response phase. Two objects were presented side by side; one was a lure and the other was a member of the previously seen set. Participants used a keyboard button to indicate whether the member of the set was displayed the right or the left side of the screen. The location (right or left) of the correct member was randomized throughout the experiment. Participants' memory capacity was estimated using the following formula: MC=*I* × *P*, where MC represents working memory capacity, I represents the number of items in the set, and P represents (proportion correct − 0.50) × 2.

### Data availability

All relevant data are available from the authors.

## Additional information

**How to cite this article**: Leib A. Y. *et al*. Fast ensemble representations for abstract visual impressions. *Nat. Commun.* 7:13186 doi: 10.1038/ncomms13186 (2016).

## Supplementary Material

Supplementary Information Supplementary Figures 1-4, Supplementary Tables 1 and 2 and Supplementary References

## References

[b1] Noë A., Pessoa L. & Thompson E. Beyond the grand illusion: what change blindness really teaches us about vision. Vis. cogn. 7, 93–106 (2000).

[b2] Alvarez G. A. & Oliva A. Spatial ensemble statistics are efficient codes that can be represented with reduced attention. Proc. Natl Acad. Sci. USA 106, 7345–7350 (2009). 19380739 10.1073/pnas.0808981106 PMC2670879

[b3] Parkes L., Lund J. & Angelucci A. Compulsory averaging of crowded orientation signals in human vision. Nat. Neurosci. 4, 739–744 (2001). 11426231 10.1038/89532

[b4] Watamaniuk S. N. J. & Duchon A. The human visual system averages speed information. Vision Res. 32, 931–941 (1992). 1604862 10.1016/0042-6989(92)90036-i

[b5] Haberman J. & Whitney D. Rapid extraction of mean emotion and gender from sets of faces. Curr. Biol. 17, 751–753 (2007). 10.1016/j.cub.2007.06.039 PMC3849410 17803921

[b6] Sperber D., Premack D. & Premack A. J. (eds). *Causal cognition: A multidisciplinary debate*. (Clarendon Press, Oxford, UK, 1995).

[b7] Heberlien A. S. in Understanding events: From perception to action (eds Shipley, T. & Zacks, J.) 363–388Oxford University Press, New York, NY, USA, (2008).

[b8] Lang P. J., Bradley M. M. & Cuthbert B. N. International Affective Picture System (IAPS): Technical Manual and Affective Ratings. (National Institute of Mental Health Center for the Study of Emotion and Attention, Gainesville, FL, 1997).

[b9] Dasser V., Ulbaek I. & Premack D. The Perception of Intention. Science 243, 365–367 (1989). 2911746 10.1126/science.2911746

[b10] Baker C., Saxe R. & Tenenbaum J. Action understanding as inverse planning. Cognition 113, 329–349 (2015). 10.1016/j.cognition.2009.07.005 19729154

[b11] Tavares P., Lawrence A. D. & Barnard P. J. Paying attention to social meaning: an fMRI study. Cereb. Cortex 18, 1876–1885 (2008). 18065722 10.1093/cercor/bhm212

[b12] New J., Cosmides L. & Tooby J. Category-specific attention for animals reflects ancestral priorities, not expertise. Proc. Natl Acad. Sci. USA 104, 16598–16603 (2007). 17909181 10.1073/pnas.0703913104 PMC2034212

[b13] Vrticka P., Sander D. & Vuilleumier P. Influence of adult attachment style on the perception of social and non-social emotional scenes. J. Soc. Pers. Relat. 29, 530–544 (2012).

[b14] Grühn D. & Scheibe S. Age-related differences in valence and arousal ratings of pictures from the International Affective Picture System (IAPS): do ratings become more extreme with age? Behav. Res. Meth. 40, 512–521 (2008). 10.3758/brm.40.2.512 18522062

[b15] Schupp H. T. . Affective picture processing: the late positive potential is modulated by motivational relevance. Psychophysiology 37, 257–261 (2000). 10731776

[b16] Haberman J. & Whitney D. in From perception to consciousness: Searching with Anne Treisman (eds Wolfe, J. & Robertson, L.) 339–349Oxford University Press, New York, NY (2012).

[b17] Mar R. A. & Macrae C. N. in Empathy and Fairness Novartis Foundation Symposium 278 (eds Bock, G. & Goode, J.) 111–132John Wiley and Sons, Chichester, UK (2012).

[b18] Chong S. C. & Treisman A. Representation of statistical properties. Vision Res. 43, 393–404 (2003). 12535996 10.1016/s0042-6989(02)00596-5

[b19] Haberman J. & Whitney D. Seeing the mean: ensemble coding for sets of faces. J. Exp. Psychol. Hum. Percept. Perform. 35, 718–734 (2009). 19485687 10.1037/a0013899 PMC2696629

[b20] Sweeny T. D., Haroz S. & Whitney D. Perceiving group behavior: sensitive ensemble coding mechanisms for biological motion of human crowds. J. Exp. Psychol. Hum. Percept. Perform. 39, 329–337 (2013). 22708744 10.1037/a0028712

[b21] McGraw K. O. & Wong S. P. Forming inferences about some intraclass correlation coefficients. Psychol. Meth. 1, 30–46 (1996).

[b22] Cicchetti D. V. Guidelines, criteria, and rules of thumb for evaluating normed and standardized assessment instruments in psychology. Psychol. Assessment 6, 284 (1994).

[b23] Piazza E. A, Sweeny T. D., Wessel D., Silver M. A. & Whitney D. Humans use summary statistics to perceive auditory sequences. Psychol. Sci. 24, 1389–1397 (2013). 23761928 10.1177/0956797612473759 PMC4381997

[b24] Yamanashi Leib A. . Ensemble crowd perception: a viewpoint-invariant mechanism to represent average crowd identity. J. Vis. 14, 1–13 (2014). 10.1167/14.8.26 PMC4114593 25074904

[b25] Hubert-wallander B. & Boynton G. M. Not all summary statistics are made equal: evidence from extracting summaries across time. J. Vis. 15, 1–12 (2015). 10.1167/15.4.5 PMC4463776 26053144

[b26] Haberman J. & Whitney D. Averaging facial expression over time. J. Vis. 9, 1–13 (2009). 10.1167/9.11.1 PMC2857387 20053064

[b27] Albrecht A. R. & Scholl B. J. Perceptually averaging in a continuous visual world: extracting statistical summary representations over time. Psychol. Sci. 21, 560–567 (2010). 20424102 10.1177/0956797610363543

[b28] Haberman J. & Whitney D. Efficient summary statistical representation when change localization fails. Psychon. Bull. Rev. 18, 855–859 (2011). 21748419 10.3758/s13423-011-0125-6 PMC3627736

[b29] Alvarez G. A. & Oliva A. The representation of simple ensemble visual features outside the focus of attention. Psychol. Sci. 19, 392–398 (2008). 18399893 10.1111/j.1467-9280.2008.02098.x PMC2587223

[b30] Ariely D. Seeing sets: representation by statistical properties. Psychol. Sci. 12, 157–162 (2001). 11340926 10.1111/1467-9280.00327

[b31] Koldewyn K., Hanus P. & Balas B. Visual adaptation of the perception of ‘life': animacy is a basic perceptual dimension of faces. Psychon. Bull. Rev. 21, 969–975 (2014). 24323739 10.3758/s13423-013-0562-5 PMC4051862

[b32] Pavlova M. & Sokolov A. Prior knowledge about display inversion in biological motion perception. Perception 32, 937–946 (2003). 14580140 10.1068/p3428

[b33] Reed C. L., Stone V. E., Bozova S. & Tanaka J. The body-inversion effect. Psychol. Sci. 14, 302–308 (2016). 10.1111/1467-9280.14431 12807401

[b34] Li FF., VanRullen R., Koch C. & Perona P. Rapid natural scene categorization in the near absence of attention. Proc. Natl Acad. Sci. USA 99, 9596–9601 (2002). 12077298 10.1073/pnas.092277599 PMC123186

[b35] Rousselet G. A, Fabre-Thorpe M. & Thorpe S. J. Parallel processing in high-level categorization of natural images. Nat. Neurosci. 5, 629–630 (2002). 12032544 10.1038/nn866

[b36] Sha L. . The animacy continuum in the human ventral vision pathway. J. Cogn. Neurosci. 27, 1–14 (2015). 25269114 10.1162/jocn_a_00733

[b37] Carruthers P. & Smith P. Theories of Theories of Mind Cambridge University Press (1996).

[b38] Blakemore S.-J. & Frith C. The role of motor contagion in the prediction of action. Neuropsychologia 43, 260–267 (2005). 15707910 10.1016/j.neuropsychologia.2004.11.012

[b39] Keysers C. & Perrett D. I. Demystifying social cognition: a Hebbian perspective. Trends Cogn. Sci. 8, 501–507 (2004). 15491904 10.1016/j.tics.2004.09.005

[b40] Scholl B. J. & Tremoulet P. D. Perceptual causality and animacy. Trends Cogn. Sci. 4, 299–309 (2000). 10904254 10.1016/s1364-6613(00)01506-0

[b41] Shor R. Effect of preinformation upon human characteristics attributed to animated geometric figures. J. Abnorm. Sociol. 54, 124–126 (1957). 10.1037/h0045604 13405667

[b42] Schlottmann A. & Anderson N. H. An information integration approach to phenomenal causality. Mem. Cognit. 21, 785–801 (1993). 10.3758/bf03202746 8289656

[b43] Brady T. F., Konkle T., Alvarez G.A. & Oliva A. Visual long-term memory has a massive storage capacity for object details. Proc. Natl Acad. Sci. USA. 105, 14325–14329 (2008). 18787113 10.1073/pnas.0803390105 PMC2533687

[b44] Brainard D. H. The psychophysics toolbox. Spat. Vis. 10, 433–436 (1997). 9176952

[b45] Kleiner M., Brainard D. & Pelli D. What's new in Psychotoolbox-3? Percept. 36 ECVP Abstr. Suppl. 1–16 (2007).

[b46] Pelli D. The video toolbox software for visual psychophysics: Transforming numbers into movies. Spat. Vis. 10, 447–432 (1997). 9176953

