# Supplementary material for: Fast ensemble representations for abstract visual impressions
Source: Nat Commun. 2016 Nov 16;7:13186. doi: 10.1038/ncomms13186 (PMC5116093; doi:10.1038/ncomms13186)
Supplement: Supplementary Information — Supplementary Figures 1-4, Supplementary Tables 1 and 2 and Supplementary References [file ncomms13186-s1.pdf]

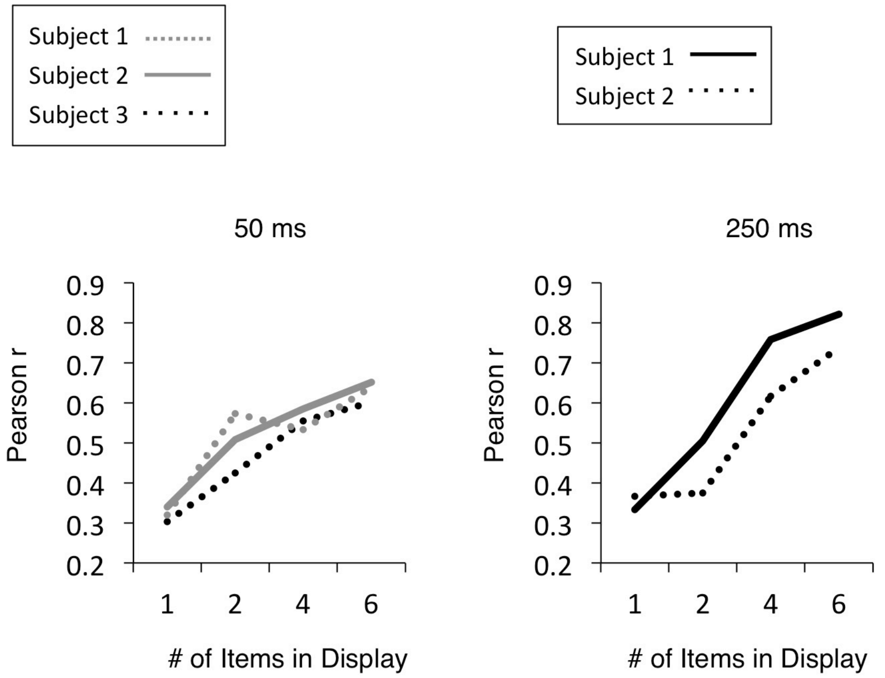

Supplementary Figure 1: Experiments 1 & 2, Integration after backward masking

To limit visual processing time, we reran two display durations in Experiment 3 with the addition of a backward mask. Stimuli were presented for either 50 or 250 ms (blocked). The mask was presented following the stimulus with 0 ISI and a duration that matched that of the display (50 or 250 ms). Observers (n = 3, n = 2) completed 152 trials in each condition (subsets 1, 2, 4, & whole set condition 6), totaling 608 trials for each display duration. The results were very similar to the results in Experiments 2-6 presented in the main text. Participants showed a strong subset effect during both exposure conditions, suggesting integration of most or all display items. The participants' Fisher z scores in each exposure duration condition were well fit by a linear model (250 ms condition linear model r^2 = .877, p = 0.001, n = 12; 50 ms condition linear model r^2 = .789, $p < 0.001$, n = 8). Supplementary Table 2 quantifies how many display items were integrated by subjects.

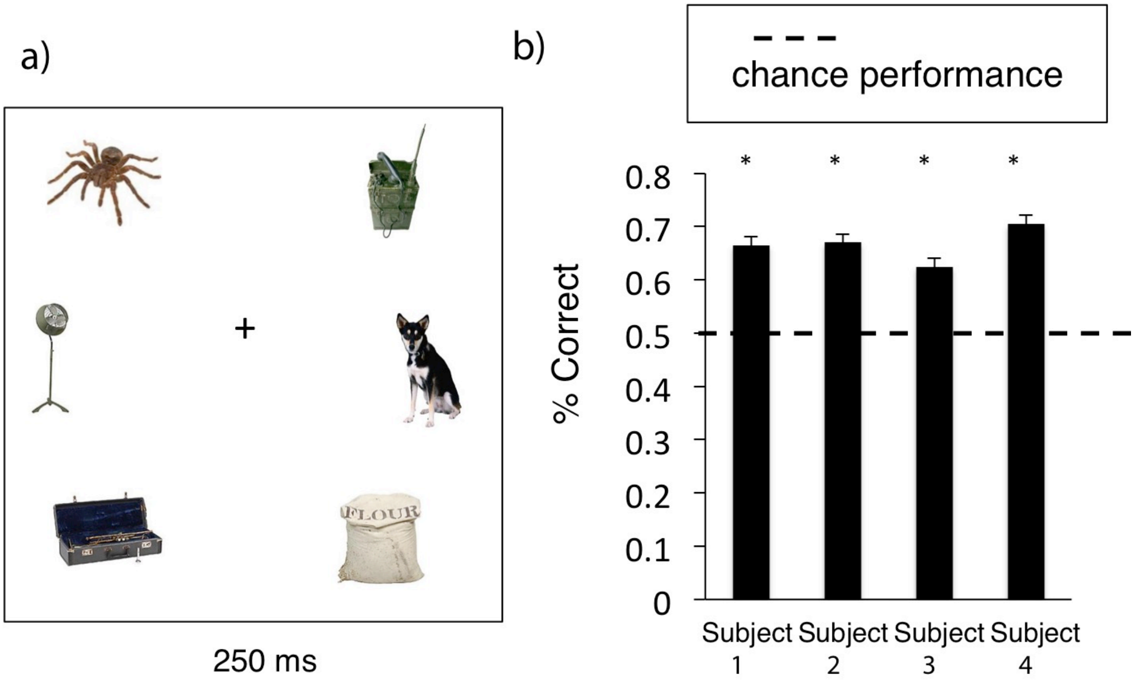

Supplementary Figure 2. Supplementary Experiment 3: Binary vs. graded animacy
To rule out a binary strategy, we ran an additional control experiment. A) In each trial, participants viewed 6 stimuli for 250 ms, displayed isoeccentrically from a fixation cross. Each side (left versus right) of the display contained the same number of animate objects (stimuli rated above '5' by observers in Experiment 1). After the display disappeared, participants were asked to report which side of the display exhibited the highest average lifelikeness. Each participant completed 200 trials. Because the number of animate objects was equated on both sides of the display, any strategy that involves binary estimates, or counting the number of animate objects, would make the two sides of the display perceptually indiscriminable. Although the absolute number of animate objects is equated, there was, on average, a very small difference in the average animacy between the two halves of the screen (on average, a 1.19 predicted lifelikeness rating difference). This is a small difference (see the x axis on Figures 2b of the main text for reference) and obviously would make the 2AFC discrimination task difficult. Nonetheless, if subjects perceive graded differences in animacy, above chance performance should be observed. **B)** All participants performed significantly above chance, $p < 0.001$, $n = 200$, as measured by a binomial test. Thus, we can be confident that participants did not solely rely on a binary strategy. We also confirmed that the individual participants agreed with independent observers (from Experiment 1) 99.33% of the time. As a further control, we excluded any stimuli that participants disagreed on, and repeated the analysis. The results were similar. All participants performed significantly above chance (binomial test; S1, $p < 0.001$, $n = 199$; S2, $p < 0.001$, $n = 200$; S3, $p < 0.006$, $n = 198$; S4, $p < 0.001$, $n = 199$), suggesting that participants relied on a graded strategy.

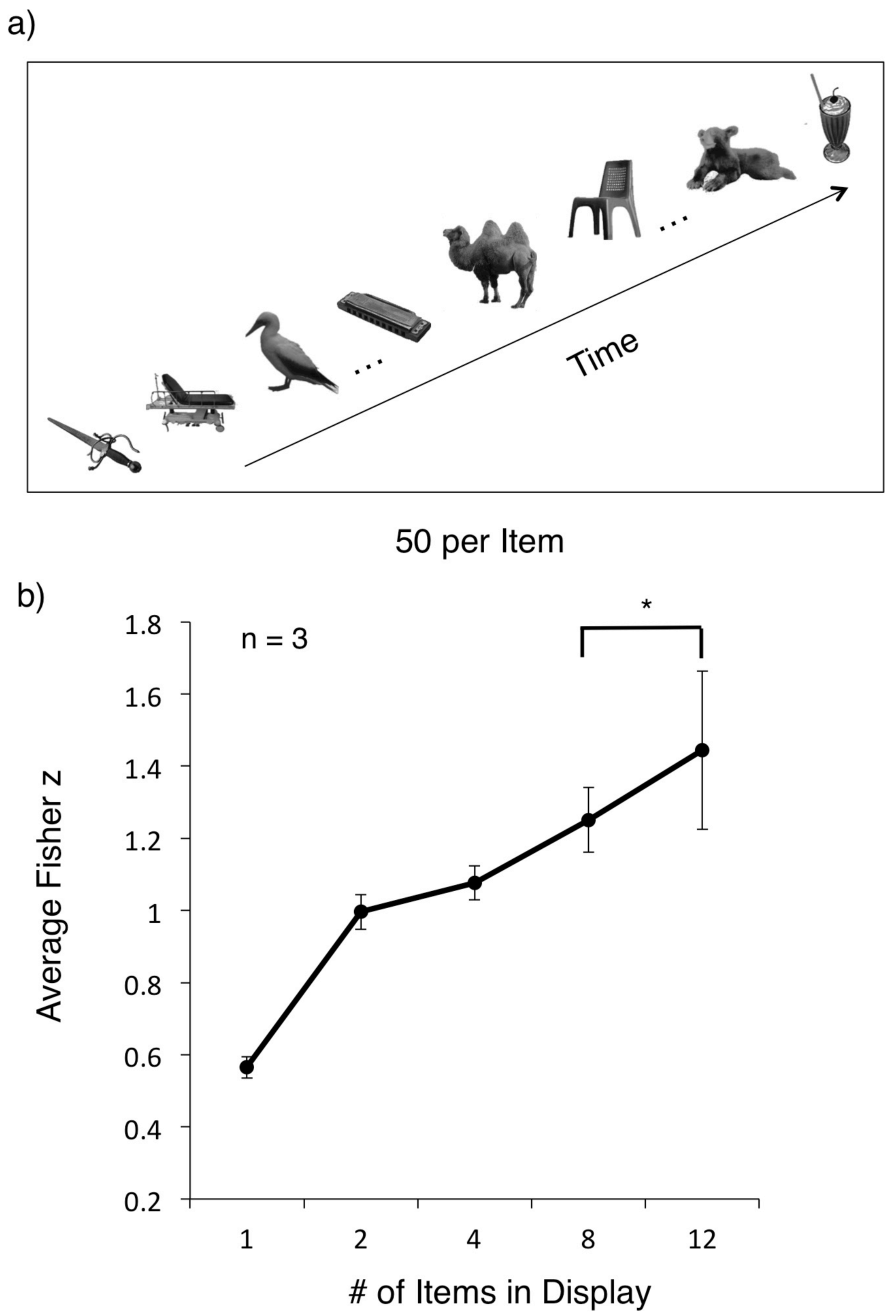

Supplementary Figure 3. Supplementary Experiment 4: Low-level feature control
A) To ensure that low-level qualities, such as the color and luminance of the stimuli, did not drive the observed ensemble coding of lifelikeness, we repeated Experiment 6 in the main text using grayscale stimuli with equated luminance histograms. Participants viewed a sequentially presented set of up to 12 stimuli. Each stimulus was presented for 50 ms with a 50 ms ISI. After the group of sequentially presented stimuli disappeared, participants rated the average lifelikeness of the group on a Likert scale, with 1 representing the lowest lifelikeness and 10 representing the highest lifelikeness. Participants were not given a time limit to make their response. Just as before, we included subsets in the experimental design to measure integration. In the subset conditions, participants viewed each item for a longer duration to equalize the total visible stimulus duration

(See Methods). **B)** The results were consistent with the findings from Experiments 2-6. In the whole set condition, we observed a highly significant correlation between participants' lifelikeness ratings of the group and the predicted lifelikeness ratings, Fisher Z = 1.44, $r_{z'}$, $p< 0.001$, n = 3. This indicates that participants successfully extracted the average lifelikeness of the sequentially presented group. Moreover, these judgments were not based on low-level features such as color or luminance. The Fisher z scores of the participants were well fit by a linear model, with the magnitude of the correlations increasing as subset size increased, $R^2 = .63$, $p < 0.001$, n = 15, confirming that participants integrated multiple items into their ensemble percept. A permutation test comparing participants' Fisher Z values between the 8-stimulus subset and the whole (12 stimulus) set revealed a significant difference, p = .05, n = 3, with participants exhibiting a higher correlation in the whole set than in the 8-object subset. Error bars represent s.e.m.

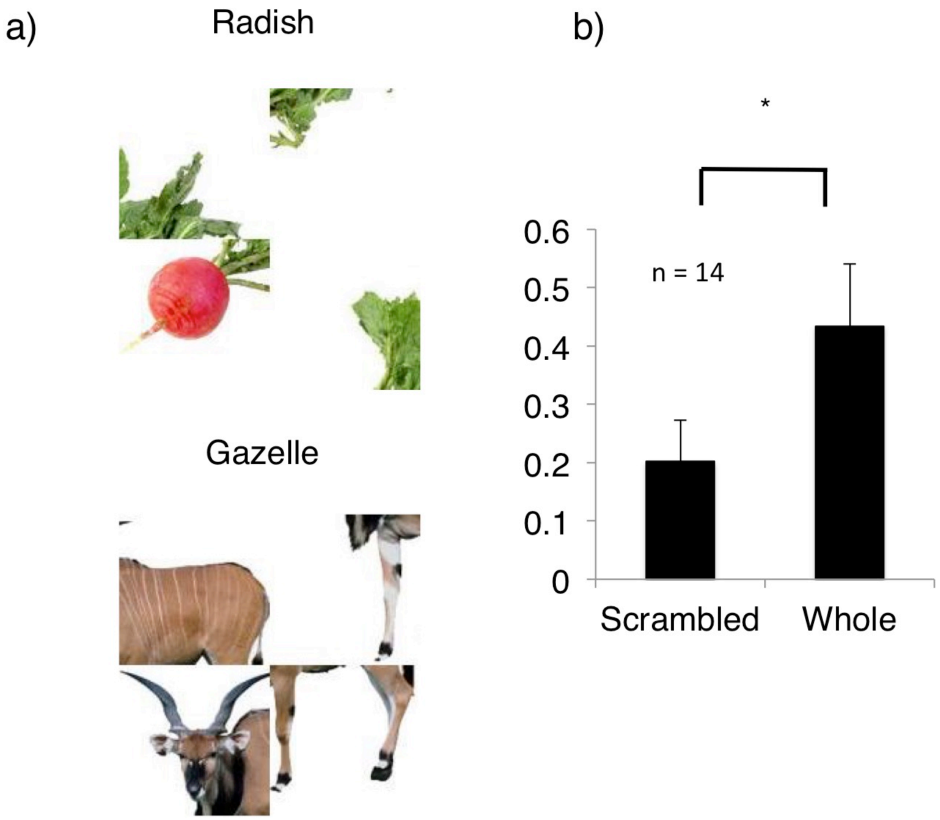

Supplementary Figure 4. Supplementary Experiment 5: Additional low-level feature control
It has been suggested that animacy perception involves configural processing [1–3], which would require that target objects be intact; spatially scrambled images should disrupt animacy perception. We scrambled the images used in all the previous experiments and retested ensemble animacy perception. **A)** Participants fixated, and viewed 6 stimuli for 250 ms, displayed isoeccentrically from a fixation cross in each trial. The 6 stimuli were either whole intact pictures or scrambled pictures. To create the scrambled stimuli, we segmented each stimulus into four quadrants and rearranged the quadrants semi-randomly (the only constraint placed on the random arrangement

was that adjacent quadrants in the original whole photo should not remain contiguous). Importantly, many of the low-level features remain intact, but configural processing is disrupted. Fourteen observers on Mechanical Turk participated in the experiment. Each participant viewed 100 trials of whole, intact stimuli and 100 trials of scrambled stimuli, totaling 200 trials. The whole and scrambled trials were randomly interleaved. After each display disappeared, participants were asked to rate the average group lifelikeness using a Likert scale from 1-10, with 1 representing low-lifelikeness and 10 representing high lifelikeness. Participants were not given a time limit to enter their response. B) We correlated participants' responses (their assessments of average group lifelikeness) with the predicted values of group lifelikeness (taken from the independent raters in Experiment 1, main text). Importantly, participants performed significantly better in the whole condition compared to the scrambled condition, t (13) = 4.002, p = 0.002, n = 14 indicating that participants relied on configural information to evaluate group lifelikeness. Error bars represent s.e.m.

**250 ms Display Duration**<br>Standardized Betas, P Values				
	Subject 1	Subject 2	Subject 3	Subject 4
Display Item 1	.256, p <0.001	.297, p <0.001	.315, p <0.001	.295, p <0.001
Display Item 2	.271, p <0.001	.348, p <0.001	.324, p <0.001	.265, p <0.001
Display Item 3	.238, p =0.001	.402, p <0.001	.176, p = 0.003	.353, p <0.001
Display Item 4	.221, p =0.001	.356, p <0.001	.346, p <0.001	.275, p <0.001
Display Item 5	.366, p <0.001	.387, p <0.001	.343, p <0.001	.336, p <0.001
Display Item 6	.127, p = .068	.375, p <0.001	.322, p <0.001	.304, p <0.001

Supplementary Table 1. Integration of all display items with brief exposure duration. Experiment 4 suggested that ensemble perception of lifelikeness can occur with brief display durations. The subset effect controls included in Experiments 4 indicated that participants integrated most or all of the display items. We also conducted a complementary regression analysis to quantify how each display item was weighted in each participant's response during brief exposure durations. The columns represent individual subjects; the rows represent each display item in a 6-item display. Beta weights indicate that all display items significantly contributed to the participant's responses in the 250 ms condition.

a)

**250 ms Display Standardized Betas, P Values**			
	**Subject 1**	**Subject 2**	**Subject 3**
**Display Item 1**	.230, $p<0.001$	.254, $p<0.001$	.218, $p<0.001$
**Display Item 2**	.253, $p<0.001$	.304, $p<0.001$	.292, $p<0.001$
**Display Item 3**	.243, $p<0.001$	.290, $p<0.001$	.264, $p<0.001$
**Display Item 4**	.344, $p<0.001$	.172, $p<0.001$	.217, $p<0.001$
**Display Item 5**	.359, $p<0.001$	.321, $p<0.001$	.251, $p<0.001$
**Display Item 6**	.319, $p<0.001$	.412, $p<0.001$	.333, $p<0.001$

b)

**250 ms Display Masked Standardized Betas, P Values**		
	**Subject 1**	**Subject 2**
**Display Item 1**	.347, $p<0.001$	.268, $p<0.001$
**Display Item 2**	.356, $p<0.001$	.218, $p<0.001$
**Display Item 3**	.375, $p<0.001$	.254, $p<0.001$
**Display Item 4**	.254, $p<0.001$	.307, $p<0.001$
**Display Item 5**	.357, $p<0.001$	.426, $p<0.001$
**Display Item 6**	.417, $p<0.001$	.260, $p<0.001$

Supplementary Table 2. Integration of all display items even when processing time is limited. For the data in Supplementary Experiment 1 & 2, (Supplementary Fig. 1) we conducted a regression analysis that quantified how much each display item contributed to each participant's response. Columns represent individual subjects; rows represent each display item in a 6-item display. Beta weights confirm that all display items significantly contributed to all participants' responses in both display conditions. These additional analyses complement and confirm our subset control findings (Supplementary Fig. 1). Participants integrate all or most of the items in the display, even with brief exposure durations, and backward masking.

Supplementary References

1. Koldewyn, K., Hanus, P. & Balas, B. Visual adaptation of the perception of 'life': animacy is a basic perceptual dimension of faces. *Psychon. Bull. Rev.* **21,** 969–975 (2014).
2. Pavlova, M. & Sokolov, A. Prior knowledge about display inversion in biological motion perception. **32,** 937–947 (2003).
3. Reed, C. L., Stone, V. E., Bozova, S. & Tanaka, J. The body-inversion effect. *Psychological Science.* **14,** 302–308 (2003).
